# Combining blood glucose and SpO_2_/FiO_2_ ratio facilitates prediction of imminent ventilatory needs in emergency room COVID-19 patients

**DOI:** 10.1038/s41598-023-50075-7

**Published:** 2023-12-20

**Authors:** Kazuya Sakai, Kai Okoda, Mototsugu Nishii, Ryo Saji, Fumihiro Ogawa, Takeru Abe, Ichiro Takeuchi

**Affiliations:** 1 Department of Emergency Medicine, Yokohama City University, School of Medicine, Fukuura, Kanazawa-ku, Yokohama, Kanagawa 236-0004 Japan; 2 Yokohama City University, School of Medicine, Fukuura, Kanazawa-ku, Yokohama, Kanagawa 236-0004 Japan

**Keywords:** Diseases, Infectious diseases, Viral infection

## Abstract

The increasing requirement of mechanical ventilation (MV) due to the novel coronavirus disease (COVID-19) is still a global threat. The aim of this study is to identify markers that can easily stratify the impending use of MV in the emergency room (ER). A total of 106 patients with COVID-19 requiring oxygen support were enrolled. Fifty-nine patients were provided MV 0.5 h (interquartile range: 0.3 to 1.4) post-admission. Clinical and laboratory data before intubation were collected. Using a multivariate logistic regression model, we identified four markers associated with the impending use of MV, including the ratio of peripheral blood oxygen saturation to fraction of inspired oxygen (SpO_2_/FiO_2_ ratio), alanine aminotransferase, blood glucose (BG), and lymphocyte counts. Among these markers, SpO_2_/FiO_2_ ratio and BG, which can be measured easily and immediately, showed higher accuracy (AUC: 0.88) than SpO_2_/FiO_2_ ratio alone (AUC: 0.84), despite no significant difference (DeLong test: P = 0.591). Moreover, even in patients without severe respiratory failure (SpO_2_/FiO_2_ ratio > 300), BG (> 138 mg/dL) was predictive of MV use. Measuring BG and SpO_2_/FiO_2_ ratio may be a simple and versatile new strategy to accurately identify ER patients with COVID-19 at high risk for the imminent need of MV.

## Introduction

At the end of 2019, an outbreak of a novel coronavirus (COVID-19) from Wuhan, China, quickly spread worldwide^1^. As a result, the World Health Organization (WHO) declared COVID-19 a pandemic on March 11, 2020. It has caused numerous infections and deaths, mainly in Europe and the United States. As of July 1, 2021, more than 180 million cases have been reported worldwide, with more than 4 million deaths^2^. Although the pathogenesis of COVID-19 has been elucidated and vaccines have been developed worldwide, it is yet to be eradicated.

COVID-19 has impacted healthcare delivery models worldwide. Many countries have experienced considerable difficulties with unexpected increases in cases, and several hospitals have reached or exceeded their capacities^3^. Approximately 30% of symptomatic patients unexpectedly progress to severe respiratory failure requiring hospitalization^4^. Therefore, medical resources such as intensive care unit (ICU) beds and ventilators are indispensable, but not inexhaustible. So far, many studies have developed a model to predict mortality and the need for the ventilatory management of patients with mild to moderate COVID-19^5–18^. However, no attempt has been made to easily and immediately predict in the emergency room (ER) whether an individual patient with severe COVID-19 requiring oxygen support is at imminent risk of progressing to critically ill COVID-19 requiring intubation and mechanical ventilation (MV).

In the present study, we hypothesized that few markers that can be immediately and easily measured would be helpful for prompt triage of patients with COVID-19 in the ER, and our data showed clinical utility of measuring blood glucose (BG) level and the ratio of peripheral blood oxygen saturation to fraction of inspired oxygen (SpO_2_/FiO_2_ ratio: S/F ratio) in risk stratification of imminent need for intubation or MV.

The objective of this study is to investigate the potential of BG levels and S/F ratio at admission as predictors for impending use of MV in ER patients with COVID-19.

## Results

### Baseline characteristics

**Figure 1:**
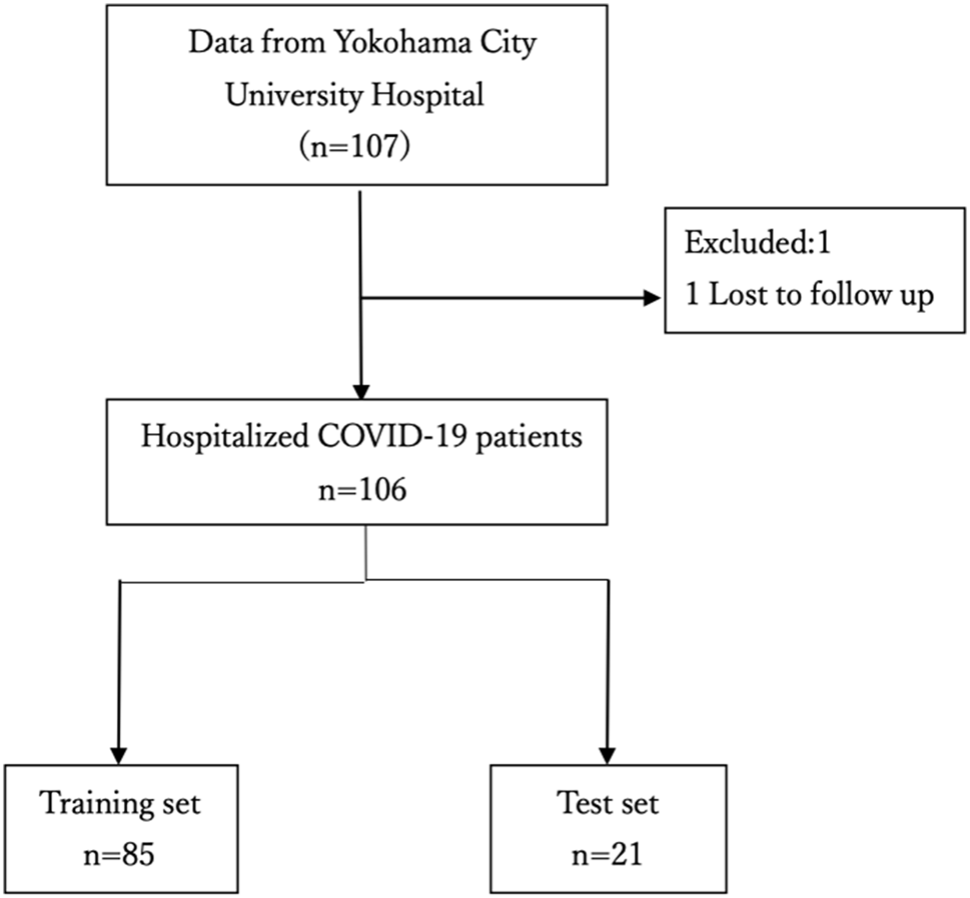
Patients flow. A total of 107 patients participated in the study. Finally, 106 patients were analyzed; 85 were used for training-data and 21 for test-data.

From February 2020 to July 2021, 107 patients with severe COVID-19 pneumonia were enrolled. Of these, 1 patient were excluded analysis because of missing data. Ultimately, a total of 106 patients were evaluated in this study (Fig. 1). Table 1 shows individual baseline clinical and outcome data in the present study population. The median age was 66 years (interquartile range [IQR]: 55 to 72). Of these patients, 77 (73%) were male and 29 (27%) were female. Hemodynamics indicated by blood pressure (BP) and heart rate (HR) was preserved at hospital arrival. More than 80% of the study population was diagnosed as pneumonia. Comorbidity was shown, including interstitial pneumonia, chronic obstructive pulmonary disease, hypertension, diabetes, chronic kidney disease, heart failure, and liver cirrhosis. More than 50% (n = 59/106) required MV for severe respiratory failure 0.5 h (IQR: 0.3 to 1.4) after hospital admission (vented group), while the remaining patients did not during hospitalization (unvented group).

**Table 1:** Clinical characteristics.

Variables	Total patients (n = 106)
Age (years)	66 [55–72]
Gender	Male:77 (72.6)
	Female:29 (27.4)
BMI	24.0 [225–27.0]
Smoking	29 (27.4)
Vital sign at hospital arrival	
SBP (mmHg)	137 [122–156]
DBP (mmHg)	81 [71–88]
BT (℃)	37 [37–38]
HR (beat/min)	90 [78–104]
RR (/min)	24 [20–28]
SpO_2_ to FiO_2_ ratio	308[117–457]
Past medical history	
IP	1 (0.9)
COPD	3 (2.8)
Asthma	13 (12.3)
HT	43 (40.6)
DM	32 (30.2)
CKD	19 (17.9)
Heart disease	9 (8.5)
HF	3 (2.8)
Dialysis	16 (15.1)
LC	7 (6.6)
Outcome data	
Mechanical ventilation	59 (56)
Hours until intubation after hospital admission (hours)	0.5 [0.3–1.4]

All categorical variables were presented as n (%). Continuous variables are shown as median values and [interquartile ranges]. BMI, body mass index; SBP, systolic blood pressure; DBP, diastolic blood pressure; BT, body temperature; HR, heart rate; RR, respiratory rate; SpO_2,_ peripheral oxygen saturation; IP, interstitial pneumonia; COPD, chronic obstructive pulmonary disease; HT, hypertension; DM, diabetes; CKD, chronic kidney disease; HF, heart failure; LC, liver cirrhosis.

### Comparisons of clinical and laboratory data

There were not any significant differences of age, sex, body mass index (BMI), and smoking between vented group and unvented group. With regarding to hemodynamics at hospital arrival, BP and HR did not show any significant differences between the 2 groups. However, respiratory status, as indicated by S/F ratio, was significantly worse before intubation in the vented group compared with in the unvented group. Alternatively, there were no significant differences in the prevalence of comorbidities between the two groups. Comparisons of laboratory data between the 2 groups showed significant increases in aspartate aminotransferase (AST), alanine aminotransferase (ALT), alkaline phosphatase (ALP), lactate dehydrogenase (LDH), blood urea nitrogen (BUN), C-reactive protein (CRP), and BG and decreases in the lymphocyte counts in the vented group (Table 2).

**Table 2:** Comparisons of clinical and laboratory data.

Variables	Unvented (n = 47)	Vented (n = 59)	P value
Age (years)	66 [49–73]	65 [57–72]	0.98
Gender	male:31 (66.0)	male:46 (78.0)	0.17
	female:16 (34.0)	female:13 (22.0)	
BMI	23.9 [21.8–26.8]	24.3 [22.7–27.8]	0.63
Smoking	11 (23.4)	18 (30.5)	0.41
Vital sign at hospital arrival			
SBP (mmHg)	138 [123–155]	136 [120–157]	0.75
DBP (mmHg)	81 [70–88]	80 [72–88]	0.87
BT (℃)	37.7 [37.1–38.2]	37.2 [36.7–37.8]	0.05
HR (beat/min)	91.5 [82.2–106.0]	85 [73–100]	0.07
RR (/min)	24 [20–25]	25 [20–30]	0.93
SpO_2_ to FiO_2_ ratio	452 [388–462]	155 [95–284]	< 0.01
Past medical history			
IP	1 (2.1)	0 (0)	0.26
COPD	2 (4.3)	1 (1.7)	0.43
Asthma	4 (8.5)	9 (15.3)	0.29
HT	15 (31.9)	28 (47.5)	0.11
DM	12 (25.5)	20 (33.9)	0.35
CKD	9 (19.1)	10 (16.9)	0.77
Heart disease	6 (12.8)	3 (5.1)	0.16
HF	1 (2.1)	2 (3.4)	0.67
Dialysis	9 (19.1)	7 (11.9)	0.30
LC	3 (6.4)	4 (6.8)	0.93
Laboratory data			
Lymphocyte (/μL)	785.5 [591.2–1087.7]	535.0 [304.1–777.5]	< 0.01
Red blood cell (×10^6^/μL)	4.34 [3.98–4.78]	4.31 [3.93–4.73]	0.70
Platelet (×10^3^/μL)	174.0 [125. 5–209.0]	191.0 [154.0–253.5]	0.14
Hemoglobin (g/dL)	13.6 [12.2–15.0]	13.4 [12.1–14.4]	0.31
PT-INR	1.11 [1.04–1.24]	1.21 [1.10–1.28]	0.05
APTT (s)	34.0 [30.8–36.0]	34.0 [31.2–37.4]	0.50
FDP-D dimer (μg/mL)	0.91 [0.50–3.05]	1.24 [0.86–2.62]	0.96
Total bilirubin (mg/dL)	0.6 [0.4–0.9]	0.5 [0.4–0.8]	0.98
AST (U/L)	31.0 [21.5–42.0]	41.0 [32.5–69.0]	< 0.01
ALT (U/L)	19.0 [15.0–29.5]	41.0 [19.0–63.5]	< 0.01
ALP (U/L)	104 [75–195]	83 [59–125]	0.04
LDH (U/L)	261.0 [208.5–346.5]	434.0 [355.5–517.0]	< 0.01
CK (U/L)	87.0 [61.5–204]	106.0 [56.0–246.5]	0.83
BUN (mg/dL)	16 [13–30]	22 [17–31]	0.04
Creatinine (mg/dL)	0.91 [0.71–1.88]	0.78 [0.63–1.16]	0.06
e-GFR (mL/min/1.73)	67.0 [28.9–85.2]	78.7 [46.2–98.0]	0.08
Na (mmol/L)	139 [136–141]	139 [136–142]	0.38
K (mmol/L)	4.0 [3.6–4.2]	4.0 [3.7–4.3]	0.26
Cl (mmol/L)	102 [100–104]	103 [99–105]	0.16
Glucose (mg/dL)	118 [106–146]	160 [138–219]	< 0.01
CRP (mg/dL)	3.7 [0.8–6.8]	9.4 [3.6–14.17]	< 0.01
Procalcitonin (ng/mL)	0.08 [0.05–0.47]	0.16 [0.07–0.33]	0.19
Troponin I (pg/mL)	11.4 [4.7–73.2]	6.4 [4.7–21.5]	0.10
CK-MB (U/L)	5 [5–5]	5 [5–5]	0.47
HbA1c (%)	6.0 [5.7–6.4]	6.3 [6.0–7.5]	0.67

All categorical variables were presented as n (%). Continuous variables are shown as median values and [interquartile ranges]. BMI, body mass index; SBP, systolic blood pressure; DBP, diastolic blood pressure; BT, body temperature; HR, heart rate; RR, respiratory rate; SpO_2,_ peripheral oxygen saturation; IP, interstitial pneumonia; COPD, chronic obstructive pulmonary disease; HT, hypertension; DM, diabetes; CKD, chronic kidney disease; HF, heart failure; LC, liver cirrhosis; PT-INR, prothrombin time-international normalized ratio; APTT, activated partial thromboplastin time; AST, aspartate aminotransferase; ALT, alanine aminotransferase; ALP, alkaline phosphatase; LDH, lactate dehydrogenase; CK, creatine kinase; BUN, blood urea nitrogen; CRP, C-reactive protein; CK-MB, creatine kinase MB.

### Prediction for the impending MV use

We evaluated the associations of significant nine variables in the univariate analysis, including S/F ratio, lymphocyte count, AST, ALT, ALP, LDH, BUN, BG, and CRP with the use of MV using the area under receiver operating characteristic curve (AUROC) obtained from a 10-split crossover test. The combination of these nine variables showed an AUC of 0.89 [0.75–1.00] with a sensitivity of 0.91, specificity of 0.81, positive predictive value (PPV) of 0.90, and negative predictive value (NPV) of 0.83 (Table 3).

**Table 3:** Receiver operating characteristic analyses for association of combined nine variables with mechanical ventilation use.

Variables	AUC	Sensitivity	Specificity	NPV	PPV
S/F, Lym, AST, ALT, ALP, LDH, BUN, BG, and CRP	0.89 [0.75–1.00]	0.91	0.81	0.83	0.90

AUC, area under the curve; NPV, Positive Negative Value; PPV, Positive Predictive Value; S/F, SpO_2_ /FiO_2_ ratio; Lym, lymphocyte counts; AST, aspartate aminotransferase; ALT, alanine aminotransferase; ALP, alkaline phosphatase; LDH, lactate dehydrogenase; BUN, blood urea nitrogen; BG, blood glucose; CRP, C-reactive protein.

**Figure 2:**
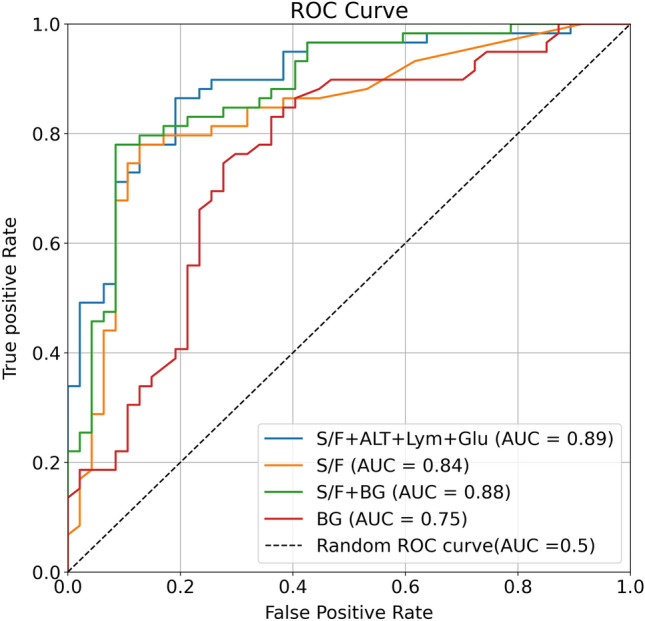
The need for MV prediction using simplified logistic regression. The area under the receiver operating characteristic curve (AUROC) of the logistic regression models (blue; S/F + Lym + ALT + BG, orange; S/F green; S/F + BG, red; BG.). S/F, SpO_2_ /FiO_2_ ratio; BG, blood glucose; Lym, lymphocyte counts; ALT, alanine aminotransferase.

The beta regression coefficient selected from a multivariable logistic regression model using these variables is shown in the supplemental Fig. 1. Based on significant beta regression coefficient with > 0.5 of their values, we identified the top four variables to predict MV use, including S/F ratio, BG, lymphocyte counts, and ALT. The combination of the four variables showed higher accuracy (AUC of 0.89 [0.83–0.95]) compared to S/F ratio or BG alone (AUC of 0.84 [0.76–0.91] or AUC of 0.75 [0.66–0.91], respectively) (Fig. 2, Table 4), with a sensitivity of 1.00, specificity of 0.82, PPV of 0.85, and NPV of 1.00 (Table 4).

**Table 4:** Receiver operating characteristic analyses for association of different four variables or combined variables with mechanical ventilation use.

Variables	AUC	Sensitivity	Specificity	NPV	PPV
S/F, BG, Lym, and ALT	0.89 [0.83–0.95]	1.00	0.82	1.00	0.85
S/F, BG	0.88 [0.82–0.94]	1.00	0.73	1.00	0.79
S/F	0.84 [0.76–0.91]	1.00	0.72	1.00	0.79
BG	0.75 [0.66–0.91]	0.63	0.63	0.64	0.64

AUC, area under the curve; NPV, Positive Negative Value; PPV, Positive Predictive Value; S/F, SpO_2_ /FiO_2_ ratio; BG, blood glucose; Lym, lymphocyte counts; ALT, alanine aminotransferase.

We further evaluated a predictive value of combing S/F ratio and BG, which can be easily and immediately measured, on the use of MV. The accuracy of this combination in the prediction (AUC: 0.88 [0.82–0.94) was nearly equal to that of the combing 4 variables, with a sensitivity of 1.00, specificity of 0.73, PPV of 0.79, and NPV of 1.00 and tended to be higher than the S/F ratio alone, despite no significant difference (Table 4). The concurrent evaluation of S/F ratio and BG is likely to allow accurately and easily predict the impending MV use in not only ER but also ambulance and home.

**Figure 3:**
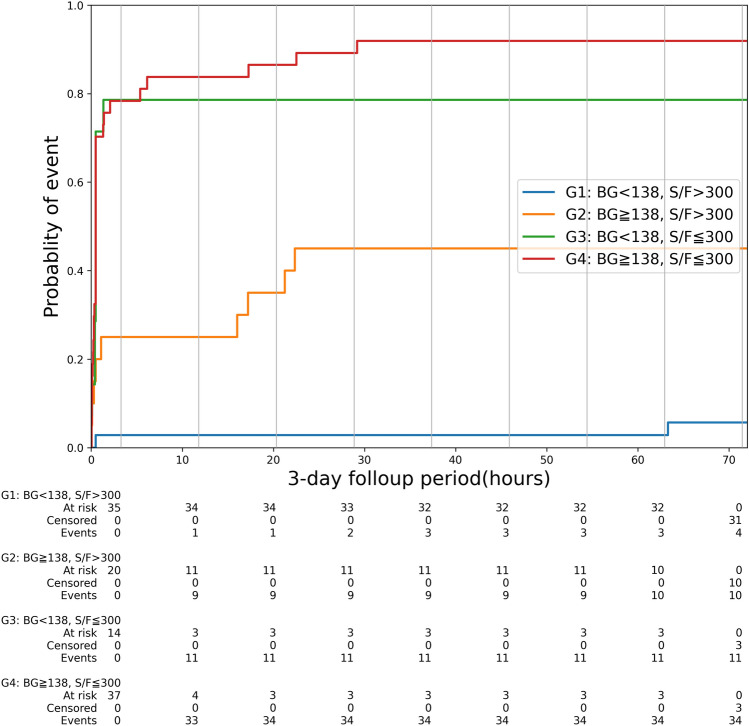
Mechanical ventilation (MV) use at the 3-day follow-up period. Kaplan–Meier event-incidence curves of MV use that were constructed according to above or below optimal cutoffs (blood glucose [BG]: 138 mg/dL, SpO_2_ /FiO_2_ ratio [S/F ratio]: 300).

Clinical utility of combining BG level and S/F ratio at hospital admission was tested with Kaplan–Meier event-incidence curves of MV use that were constructed according to above or below optimal cutoffs defined by AUROC analysis (BG: 138 mg/dL, S/F ratio: 300). In high-risk patients with low S/F ratio (≤ 300), MV use rate at the 3-day follow-up period did not show a significant difference between low BG (< 138 mg/dL) and high BG (≥ 138 mg/dL) (n = 11/14 (79%) vs. n = 34/37 (92%), respectively, P = 0.48). However, in patients with high S/F ratio (> 300), those with high BG had a significantly higher MV use rate compared to those with low BG (n = 10/20 (50%) vs. n = 4/35 (11%), respectively, P < 0.01) (Fig. 3). Importantly, this suggests that measuring BG level at hospital admission allows identifying patients at high risk for impending MV use from population with preserved respiratory status, which supports a better accuracy of combining BG and S/F ratio than S/F ratio alone.

## Discussion

Our study focused on the possibility of easily identifying ER patients with COVID-19 at high risk for the impending use of ventilation using easily and immediately measurable biomarkers at the time of admission. Our data showed for the first time that measuring BG levels and S/F ratio may facilitate the stratification of high-risk patients in the ER.

It has been demonstrated that hyperglycemia in patients with COVID-19 is associated with a higher risk of MV use or in-hospital mortality^19–23^. However, its predictive role in impending use of MV remained elusive. Presence of severe respiratory failure, as indicated by low S/F ratio, is strongly predictive of impending use of MV. Our data supported its robust predictive value. On the other hand, in our study, 25% (n = 14/55) of patients who preserved respiratory status under oxygen support (F/S ratio > 300) at hospital arrival imminently required the MV use 0.5 h post-admission, indicating the limitation of S/F ratio as a predictor in COVID-19. Importantly, our data showed that measuring BG level in the ER may allow easily identifying such high-risk patients, prior to the development of severe respiratory failure. Our study revealed for the first time the potential of BG level, which can be easily and quickly measured in the ER, as a predictor of imminent MV use.

It has been reported that hyperglycemia in COVID-19 patients is associated with a higher risk of in-hospital mortality regardless prior history or presence of diabetes^23^. In infectious diseases, secretion of catecholamines from the adrenal medulla leads to hyperglycemia, indicating increased systemic stress^24^. In turn, the increased glucose metabolism imposed by sustained hyperglycemia seems to enhance SARS-CoV-2’s entry and subsequent replication, as well as exacerbated immune responses such as tumor necrosis factor-α, interleukin (IL)-1β, and IL-6^25,26^. Thus, a disrupted glucose metabolism and metabolic derangement may be an intrinsic cellular strategy that favors SARS-CoV-2 pathogenesis^26^. Therefore, hyperglycemia may not only be a result of in vivo reactions, but it may also be involved in the enhancement of immune responses and excessive immune responses, such as those seen in COVID-19.

Our study exhibited several strengths. First, our results were comparable to those of previously reported predictive models. Second, we developed a simplified but highly predictive model to improve the clinical utility of this model and the required factors are easy to obtain and can be implemented immediately. Blood glucose can be measured with a meter developed for self-monitoring of blood glucose (SMBG) in diabetic patients, and the S/F ratio can be easily calculated from a pulse oximeter. By using these simple measurement devices, these variables are possible to measure outside the hospital. Therefore, in a pandemic setting where adequate medical care cannot be supplied, this model can be used to screen for severe disease in places such as homes and clinics without adequate medical equipment.

This study has several limitations. Because our study dataset was a backward-looking study, only the parameters listed in the electronic medical records could be analyzed, and there were several missing values. In addition, because it was a single-center study, the sample size in our study was small. Thus, we could not evaluate the generalizability of the machine learning model. Further research in a larger cohort across multiple centers is needed to determine the applicability of the newly developed machine learning models and validate the significance of our findings in clinical practice. In addition, the race of the patients was biased toward the Japanese. Because previous literature has reported that there were racial differences in the severity of COVID-19 cases, it is necessary to verify whether the developed model can be applied in countries other than Japan. The blood glucose value used in this study was measured at the hospital, not the blood glucose value measured by the meter for SMBG. Past studies have shown that capillaries correlate with plasma glucose levels, and the results of this study may be applicable to simple blood glucose meters^27^. However, approximately 70% of the study population was non-diabetic. Therefore, further studies are needed to explore the potential utility of BG measurement in diabetic patients at high risk for COVID-19 deterioration. In this study population, the proportion of patients requiring MV was 50%, notably higher than the reported worldwide average of 25% for severe COVID-19 cases. This discrepancy may reflect a combination of factors, such as the inclusion of non-invasive ventilators, the patient population during the pandemic period when therapeutic agents had not yet been established, and our facility's location in a large metropolitan area.

In conclusion. our data suggest that measuring both BG and SpO_2_/FiO_2_ ratio may be a new simple and versatile strategy to easily identify ER patients with COVID-19 at high risk for the imminent MV need.

## Methods

### Study design

This was a retrospective observational study conducted at one hospital in Japan. Patients with severe COVID-19 pneumonia requiring oxygen support who were admitted to the Yokohama City University Hospital (YCUH) between February 2020 and July 2021 were enrolled in this study. COVID-19 pneumonia was diagnosed by via Polymerase Chain Reaction (PCR) and chest Xp. Enrolled patients were observed during 30-day after the enrollment to evaluate clinical outcome. The primary outcome was the use of MV. Patients with missing data, those who offered to withdraw from the study, pregnant women, age < 20 years, and those who did not require oxygen support in the ER were excluded from the evaluation. With complete clinical, laboratory, and outcome data and oxygen support in the ER as well as consent for participation, a total of 106 COVID-19 patients were included in the final analysis.

### Ethical considerations

This study was performed in accordance with the Helsinki Declaration was approved by the Institutional Ethics Board of the Yokohama City University Hospital (No. B210100010). All research was performed in accordance with the relevant guidelines and regulations. During hospitalization, patients were provided negative and positive information regarding this study, including the purpose and contribution of this study, the use of personal information, and complications associated with blood collection, and were asked to participate in this study. Ultimately, we obtained written informed consent for participation in the study and access to medical and laboratory records from patients. The study had no risk/negative consequence on those who participated in the study. Medical record numbers were used for data collection and no personal identifiers were collected or used in the research report. Data was accessed from February 16, 2020, to July 5, 2021, and access to the collected information was limited to the principal investigator and confidentiality was maintained throughout the project.

### Data and specimen collection

We obtained clinical and laboratory in the ER, and treatment and outcome data were obtained from electronic medical records. Two researchers independently reviewed the data collection forms to double-check the collected data.

### Definition of severity by COVID-19

The illness severity of COVID-19 was defined according to the Ministry of Health, Labor, and Welfare in Japan, which defines critically ill patients as those requiring ventilators or treatment in intensive care units^28^. The need for MV was determined by the ratio of the partial pressure of arterial oxygen to FiO_2_ (PaO_2_/FiO_2_) and respiratory pattern; a MV was introduced if PaO_2_/FiO_2_ was less than 200 or if PaO_2_/FiO_2_ was less than 250 and excessive effort breathing continued.

### Development of the model

The following methods were used to develop the predictive model: (1) data preprocessing and (2) variable selection and model evaluation.

#### Data preprocessing

Covariates missing more than 5% of their data were excluded. In the case of highly correlated pairs of variables (correlation coefficient > 0.8), a variable with a higher missing rate were removed. For missing values, median values were imputed.

#### Variable selection and model evaluation

The data set was divided into vented and non-vented groups. The variables included in the univariate and multivariate analysis were selected based on their clinical relevance and reported importance in previous literature. Following the univariate analysis, variables that showed a statistically significant association with the outcome of interest were further included in the multivariate logistic regression model to adjust for potential confounders. The beta regression coefficient selected from this model is shown in the supplemental Fig. 1. We selected common components of these models, based on significant beta regression coefficient with > 0.5 of their values. A beta coefficient > 0.5 was chosen for inclusion in the model. While this threshold was an arbitrary decision based on our previous experience, it typically indicates a relatively strong association in the context of our logistic regression model. Next, we developed a simplified prognostic model using an interaction from two variables (S/F ratio, BG), because they were easy to use in clinical practice. We evaluated the performances of the models using the area under the receiver operating curve, AUROC, sensitivity, and specificity values obtained via tenfold cross-validation. The sensitivity, specificity, or AUC performances were defined as poor with a value < 0.5, low with a value between 0.5 and 0.7, moderate with a value between 0.7 and 0.85, and excellent with a value > 0.85^29^.

We used Python (3.7.10) for data collection, data cleaning, functional engineering, and machine-learning training and testing. The development environment included JupyterNotebook. The main libraries included Numpy, Pandas, Sklearn, Scipyand matplotlib. To prevent overfitting, we used k-split cross-testing and hyperparameter optimization during training.

### Statistical analysis

Data analysis was performed using the Python software version 3.7.10. Dependent variables were presented as the median (IQR) for continuous variables frequencies (%) for categorical variables. Differences between the vented and non-vented groups were analyzed with Fisher's exact test for categorical data or the Mann–Whitney *U* test for continuous data. Receiver operating curve, ROC, analysis and the AUROC were used to evaluate the ability of each scoring system to predict an increase in ventilation. The AUROC were compared between groups using the DeLong test. To test an interaction between S/F ratio and BG on event-free survival, Kaplan Meier survival curve was drawn, and log-rank tests with multiple comparisons were performed. We determined p < 0.05 as statistically significant.

### Supplementary Information

Supplementary Table 1.

Supplementary Figure 1.

## Data Availability

All data generated or analyzed during this study are included in supplementary information files (supplemental Table 1).
