# Supplementary material for: Combining blood glucose and SpO2/FiO2 ratio facilitates prediction of imminent ventilatory needs in emergency room COVID-19 patients
Source: Sci Rep. 2023 Dec 20;13:22718. doi: 10.1038/s41598-023-50075-7 (PMC10733355; doi:10.1038/s41598-023-50075-7)
Supplement: Supplementary file 1 — Supplementary Table 1. [file 41598_2023_50075_MOESM1_ESM.docx]

**Supplemental Table 1. Detailed Clinical Data Used in Model Construction.**

| **Case** | **Age** | **Gender** | **DM** | **Lym(/μL)** | **ALT(U/L)** | **BG(mg/dL)** | **S/F** | **Ventilated** |
| --- | --- | --- | --- | --- | --- | --- | --- | --- |
| 1 | 86 | Male | Yes | 850.2 | 43 | 187 | 316.67 | Yes |
| 2 | 64 | Male | No | 904.8 | 41 | 131 | 133.8 | Yes |
| 3 | 66 | Male | Yes | 1066 | 22 | 106 | 457.14 | No |
| 4 | 91 | Male | No | 759 | 18 | 111 | 438.1 | No |
| 5 | 39 | Male | No | 1149.2 | 56 | 93 | 466.67 | No |
| 6 | 69 | Male | Yes | 300 | 18 | 217 | 452.38 | Yes |
| 7 | 67 | Male | No | 651 | 33 | 100 | 271.88 | Yes |
| 8 | 72 | Female | Yes | 615.6 | 16 | 181 | 447.62 | No |
| 9 | 75 | Male | No | 593.6 | 15 | 121 | 447.62 | No |
| 10 | 61 | Male | Yes | 248 | 27 | 265 | 150 | Yes |
| 11 | 49 | Male | No | 1568.8 | 74 | 100 | 461.9 | No |
| 12 | 30 | Male | Yes | 342.2 | 448 | 158 | 457.14 | Yes |
| 13 | 72 | Male | No | 1109.4 | 28 | 110 | 447.62 | No |
| 14 | 75 | Male | Yes | 446.4 | 11 | 198 | 303.13 | No |
| 15 | 29 | Male | No | 850.5 | 31 | 90 | 404.76 | No |
| 16 | 48 | Female | No | 588.8 | 19 | 114 | 471.43 | No |
| 17 | 30 | Female | No | 964.6 | 19 | 105 | 471.43 | No |
| 18 | 26 | Male | No | 2371.6 | 11 | 88 | 466.67 | No |
| 19 | 73 | Male | Yes | 785.4 | 16 | 155 | 466.67 | No |
| 20 | 80 | Male | No | 668.8 | 60 | 165 | 120 | Yes |
| 21 | 50 | Male | No | 649.3 | 105 | 173 | 90 | Yes |
| 22 | 65 | Male | Yes | 693 | 11 | 113 | 471.43 | No |
| 23 | 57 | Female | No | 450 | 27 | 114 | 466.67 | No |
| 24 | 76 | Male | No | 787.5 | 115 | 190 | 50 | Yes |
| 25 | 69 | Female | Yes | 800 | 16 | 143 | 466.67 | No |
| 26 | 54 | Male | No | 2966.5 | 35 | 124 | 457.14 | No |
| 27 | 70 | Female | Yes | 448 | 57 | 259 | 272.22 | Yes |
| 28 | 72 | Male | No | 616.4 | 56 | 103 | 438.1 | No |
| 29 | 66 | Male | Yes | 843.5 | 15 | 243 | 466.67 | No |
| 30 | 70 | Female | No | 565.5 | 19 | 107 | 461.9 | No |
| 31 | 70 | Male | No | 1018.5 | 19 | 111 | 461.9 | No |
| 32 | 78 | Male | No | 639.1 | 55 | 146 | 275 | No |
| 33 | 52 | Female | No | 276 | 57 | 176 | 156.67 | Yes |
| 34 | 66 | Male | No | 780 | 76 | 215 | 192 | Yes |
| 35 | 74 | Male | Yes | 1268 | 102 | 121 | 184 | Yes |
| 36 | 61 | Male | No | 855 | 70 | 129 | 184 | Yes |
| 37 | 65 | Male | Yes | 819 | 15 | 225 | 380 | No |
| 38 | 75 | Male | No | 489.5 | 11 | 138 | 423.81 | Yes |
| 39 | 56 | Female | Yes | 775 | 197 | 210 | 94 | Yes |
| 40 | 28 | Male | No | 1118 | 11 | 104 | 452.38 | No |
| 41 | 64 | Male | Yes | 957 | 5 | 237 | 404.17 | No |
| 42 | 72 | Male | Yes | 68.8 | 22 | 147 | 95 | Yes |
| 43 | 70 | Male | Yes | 62 | 48 | 291 | 96 | Yes |
| 44 | 79 | Female | Yes | 263.2 | 24 | 293 | 92 | Yes |
| 45 | 68 | Male | Yes | 46 | 45 | 585 | 105.56 | Yes |
| 46 | 95 | Female | No | 973.7 | 33 | 113 | 366.67 | No |
| 47 | 68 | Male | No | 969.9 | 18 | 137 | 86 | Yes |
| 48 | 69 | Male | No | 378 | 22 | 145 | 466.67 | Yes |
| 49 | 42 | Male | No | 262.5 | 85 | 138 | 95 | Yes |
| 50 | 74 | Male | No | 210 | 65 | 192 | 161.67 | Yes |
| 51 | 66 | Male | Yes | 129 | 21 | 138 | 75 | Yes |
| 52 | 59 | Male | Yes | 829.4 | 10 | 161 | 466.67 | Yes |
| 53 | 76 | Male | Yes | 1721.9 | 5 | 187 | 452.38 | No |
| 54 | 64 | Male | No | 231 | 40 | 130 | 151.67 | Yes |
| 55 | 73 | Male | Yes | 313.5 | 17 | 255 | 97 | Yes |
| 56 | 79 | Female | Yes | 706.8 | 14 | 568 | 457.14 | Yes |
| 57 | 72 | Male | Yes | 127.4 | 16 | 228 | 155 | Yes |
| 58 | 70 | Male | Yes | 735 | 48 | 219 | 100 | Yes |
| 59 | 76 | Male | Yes | 735 | 48 | 219 | 83 | Yes |
| 60 | 83 | Male | No | 660.8 | 7 | 108 | 461.9 | Yes |
| 61 | 61 | Male | No | 957 | 26 | 149 | 95 | Yes |
| 62 | 75 | Male | No | 602 | 21 | 190 | 86 | No |
| 63 | 54 | Male | Yes | 682.5 | 63 | 261 | 261.11 | Yes |
| 64 | 44 | Male | No | 330 | 48 | 135 | 65 | Yes |
| 65 | 85 | Female | No | 330 | 48 | 135 | 94 | No |
| 66 | 65 | Male | Yes | 504.7 | 12 | 123 | 457.14 | Yes |
| 67 | 68 | Male | No | 500.5 | 49 | 111 | 428.57 | No |
| 68 | 56 | Male | No | 183 | 78 | 132 | 98 | No |
| 69 | 70 | Female | No | 868 | 4 | 119 | 471.43 | No |
| 70 | 39 | Male | Yes | 1963.5 | 39 | 96 | 342.86 | No |
| 71 | 71 | Male | No | 194.4 | 19 | 237 | 457.14 | No |
| 72 | 86 | Female | No | 742.4 | 20 | 102 | 461.9 | No |
| 73 | 75 | Female | No | 566.4 | 17 | 177 | 160 | Yes |
| 74 | 91 | Female | Yes | 1393.2 | 18 | 136 | 414.29 | No |
| 75 | 86 | Male | Yes | 2115.6 | 20 | 137 | 466.67 | No |
| 76 | 42 | Female | No | 697 | 27 | 151 | 284.85 | Yes |
| 77 | 35 | Female | No | 853.6 | 23 | 128 | 457.14 | No |
| 78 | 61 | Male | No | 1212.5 | 18 | 99 | 100 | Yes |
| 79 | 52 | Male | Yes | 864.6 | 35 | 202 | 237.5 | Yes |
| 80 | 65 | Male | No | 399 | 231 | 157 | 158.33 | Yes |
| 81 | 67 | Female | No | 481.5 | 20 | 151 | 192 | Yes |
| 82 | 57 | Male | Yes | 297 | 71 | 119 | 423.81 | Yes |
| 83 | 61 | Female | No | 772.8 | 20 | 118 | 388 | No |
| 84 | 48 | Male | Yes | 518.5 | 89 | 333 | 118.75 | Yes |
| 85 | 40 | Male | No | 741 | 22 | 96 | 461.9 | No |
| 86 | 82 | Female | No | 511.2 | 10 | 283 | 180 | Yes |
| 87 | 57 | Male | Yes | 959.2 | 48 | 349 | 442.86 | Yes |
| 88 | 62 | Female | No | 616 | 22 | 148 | 99 | Yes |
| 89 | 69 | Female | No | 342 | 24 | 231 | 95 | Yes |
| 90 | 51 | Male | No | 475.2 | 64 | 146 | 190 | Yes |
| 91 | 56 | Male | Yes | 535 | 65 | 219 | 300 | Yes |
| 92 | 86 | Male | No | 308.1 | 14 | 160 | 148.33 | Yes |
| 93 | 71 | Female | No | 556.5 | 15 | 211 | 146.88 | No |
| 94 | 56 | Male | No | 1276.8 | 21 | 121 | 452.38 | No |
| 95 | 43 | Male | No | 533.6 | 21 | 112 | 452.38 | No |
| 96 | 61 | Male | Yes | 1176 | 57 | 186 | 461.9 | Yes |
| 97 | 58 | Female | Yes | 1215 | 40 | 261 | 461.9 | No |
| 98 | 73 | Female | No | 742.5 | 15 | 206 | 314.29 | No |
| 99 | 57 | Male | No | 203 | 8 | 101 | 112.5 | Yes |
| 100 | 49 | Male | No | 579.6 | 42 | 154 | 93 | Yes |
| 101 | 50 | Male | No | 170.3 | 57 | 160 | 93 | Yes |
| 102 | 57 | Female | No | 862.5 | 26 | 110 | 93 | Yes |
| 103 | 59 | Female | No | 615.4 | 17 | 127 | 391.67 | Yes |
| 104 | 18 | Male | No | 566.8 | 16 | 96 | 461.9 | No |
| 105 | 43 | Male | No | 975 | 76 | 107 | 113.75 | Yes |
| 106 | 43 | Male | No | 576.7 | 3 | 125 | 196 | No |

DM, diabetes; Lym, lymphocyte counts; ALT, alanine aminotransferase; BG, blood glucose; S/F, SpO_2_ /FiO_2_ ratio.
