# Supplementary material for: Combining blood glucose and SpO2/FiO2 ratio facilitates prediction of imminent ventilatory needs in emergency room COVID-19 patients
Source: Sci Rep. 2023 Dec 20;13:22718. doi: 10.1038/s41598-023-50075-7 (PMC10733355; doi:10.1038/s41598-023-50075-7)
Supplement: Supplementary file 2 — Supplementary Figure 1. [file 41598_2023_50075_MOESM2_ESM.pdf]

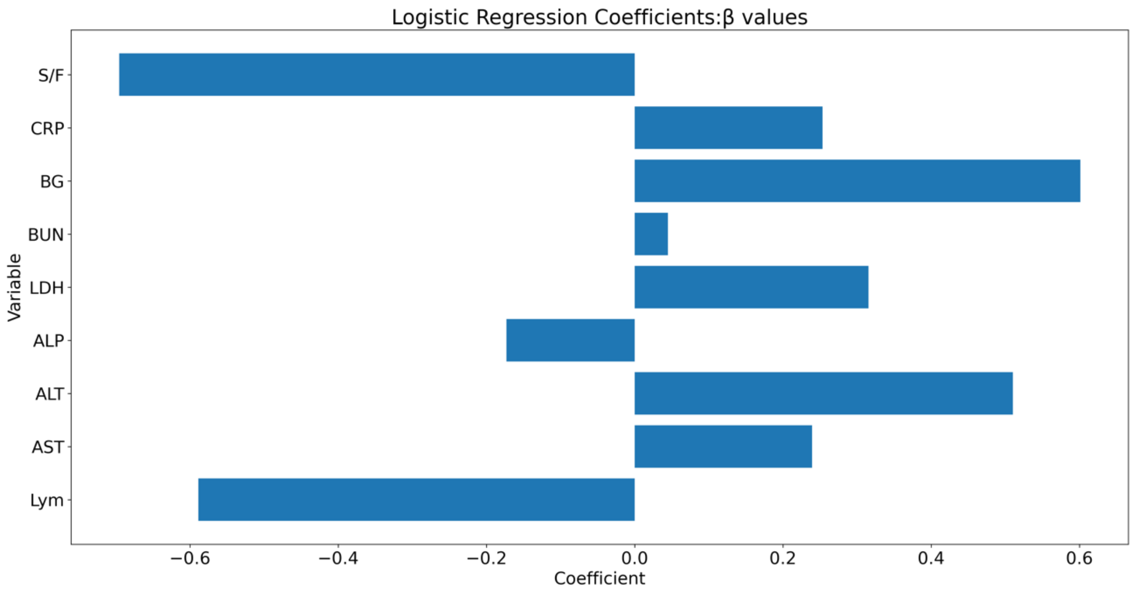

Supplementary Figure 1 Coefficients β values of logistic regression

S/F, SpO_2 /FiO_2 ratio; CRP, C-reactive protein; BG, blood glucose; BUN, blood urea nitrogen; LDH, lactate dehydrogenase; ALP, alkaline phosphatase; AST, aspartate aminotransferase; ALT, alanine aminotransferase ; Lym, lymphocyte counts.
